# Effect of storage conditions on the quality of equine and canine mesenchymal stem cell derived nanoparticles including extracellular vesicles for research and therapy

**DOI:** 10.1186/s11671-024-04026-4

**Published:** 2024-05-03

**Authors:** Michele Christian Klymiuk, Natalie Balz, Mohamed I. Elashry, Sabine Wenisch, Stefan Arnhold

**Affiliations:** 1 Institute of Veterinary-Anatomy, -Histology and -Embryology, Faculty of Veterinary Medicine, Justus-Liebig-University Giessen, Frankfurter Strasse 98, 35392 Giessen, Germany; 2 Clinic of Small Animals, c/o Institute of Veterinary-Anatomy, -Histology and -Embryology, Faculty of Veterinary Medicine, Justus-Liebig-University Giessen, Frankfurter Strasse 98, 35392 Giessen, Germany

## Abstract

Nanoparticles including extracellular vesicles derived from mesenchymal stem cells are of increasing interest for research and clinical use in regenerative medicine. Extracellular vesicles (EVs), including also previously named exosomes, provide a promising cell-free tool for therapeutic applications, which is probably a safer approach to achieve sufficient healing. Storage of EVs may be necessary for clinical applications as well as for further experiments, as the preparation is sometimes laborious and larger quantities tend to be gained. For this purpose, nanoparticles were obtained from mesenchymal stem cells from adipose tissue (AdMSC) of horses and dogs. The EVs were then stored for 7 days under different conditions (− 20 °C, 4 °C, 37 °C) and with the addition of various additives (5 mM EDTA, 25–250 µM trehalose). Afterwards, the size and number of EVs was determined using the nano tracking analyzing method. With our investigations, we were able to show that storage of EVs for up to 7 days at 4 °C does not require the addition of supplements. For the other storage conditions, in particular freezing and storage at room temperature, the addition of EDTA was found to be suitable for preventing aggregation of the particles. Contrary to previous publications, trehalose seems not to be a suitable cryoprotectant for AdMSC-derived EVs. The data are useful for processing and storage of isolated EVs for further experiments or clinical approaches in veterinary medicine.

## Introduction

Stem cells are an up-to-date method for treating various diseases. Based on the registered clinical trials in the US, myocardial infarction, graft versus host disease, diabetes, liver cirrhosis, spinal cord injury and osteoarthritis are the most common treated diseases in human medicine [1]. Especially mesenchymal stem cells (MSCs), also called medicinal signaling cells [2], which exhibit a differentiation potential to e.g. osteogenic, chondrogenic and adipogenic lineages, are most interesting for the treatment of musculoskeletal disorders [3]. In veterinary medicine MSCs are used for treatment of cartilage and tendon lesions as well as bone fractures and osteoarthritis [4, 5]. MSCs can be isolated from various types of tissues involving bone marrow, adipose tissue, umbilical blood and umbilical cord matrix. Isolation of adipose tissue derived MSCs (AdMSCs) from fat tissue or bone marrow is usually preferred, due to the comparatively easy way to obtain the necessary tissue from patients or donors followed by a fast isolation method [6, 7].

After cultivation and expansion of MSCs, various ways can be taken for their therapeutic application. Most often MSCs were expanded in vitro and applied directly to the patient, either by systemic injection or by direct injection into the region of the lesion [5, 8]. Nowadays it is known, that not only the MSCs itself, but also paracrine factors are involved in immunomodulatory effects in the recipient tissue, which may have a much higher potential to initiate regeneration than the classical hypothesis of an in situ differentiation of stem cells to replace damaged tissue for example in the musculoskeletal system [9–11].

In this aspect it has been shown that especially small extracellular vesicles (small EVs) are important in the paracrine cell–cell communication between MSCs and target cells [9, 12–14]. Small EVs are sized between 30 and 200 nm [15, 16], which originate from the endosomal membrane and were transferred in multivesicular bodies through the cytoplasm to the outer cellular membrane, where the EVs were released after fusion of the multivesicular body with the outer cell membrane [15, 17]. The EVs cargo consists of proteins, lipids, sugars, DNA/RNA derivates, mitochondrial components, cytokines and growth factor receptors to fulfill their function of intercellular communication [15, 18].

Several additives have been established to stabilize EVs. Trehalose, a non-toxic sugar, is one of the most common additives in EVs storage media, for freezing or lyophilization in order to prevent aggregation of protein containing surfaces as EVs [19, 20]. After isolation and concentration, a mixture of HEPES, albumin and trehalose also showed favorable properties for long-term storage, but also for shorter-term storage [21]. A classic cryoprotectant, dimethyl sulfoxide (DMSO), has also been used to stabilize EVs during the freezing process. However, morphological changes as well as a degradation of RNA from the EVs could be shown, which suggests a reduced biological activity [22]. Blood collection tubes treated with acidic citrate dextrose have been shown not to be cryoprotective, but to stabilize plasma EVs for further analysis [23].

However, short-term storage of fresh isolated EVs in particular can also be of great benefit. Especially if a large quantity of them has been obtained and cannot be processed immediately. Here, suitable storage must be possible in order to maintain the quality, for example for a variety of (large-scale) experiments and also for clinical use. For this purpose, we are not only testing an already commonly used sugar—trehalose, but also an anticoagulant ethylenediaminetetraacetic acid (EDTA) known from the clinic. For these investigations, the focus was on quantifying the concentration and size of nanoparticles after supplementation of either (EDTA) or trehalose (TRE) during freezing and thawing. In an additional experiment, EDTA was added to EVs stored at different temperatures (4 °C, 20 °C and 37 °C) for up to 7 days. Our data provide important insights into the processing and storage of AdMSC-derived nanoparticles for clinical approaches in veterinary medicine.

## Material and methods

### Isolation of adipose derived mesenchymal stem cells

Adipose tissue was collected from freshly slaughtered horses at the local abattoir. The collection procedure from the region above the dorsal gluteal muscle has been described elsewhere [24]. Canine adipose tissue was collected during surgical procedures unrelated to our study on dogs at the Clinic for Small Animals, Faculty of Veterinary Medicine, Justus-Liebig-University Giessen, Germany. Specimens from surgical procedures in which a toxic or infectious event was present or suspected were not used for the study. The isolation procedure to obtain AdMSCs was previously described in detail by Raabe et al. [24]. In brief, the fat obtained was minced into approximately 125 mm^3^-sized pieces and washed three times with PBS and then incubated for 1 h at 37 °C for digestion with 0.1% collagenase type I and 1% bovine serum albumin in a water bath with constant shaking. The mixture was thereafter centrifuged at 240 g for 5 min. The pellet was neatly filtered through a 70 µm filter in DMEM low glucose and transferred to cell culture. Stemness was previously verified by adherence, trilineage differentiation into adipogenic-, chondrogenic- and osteogenic lineage as described before [25]. In addition, flow cytometric analysis was used to test the identity of equine and canine AdMSCs for the typical expression of CD90^+^, CD44^+^, CD45^−^, MHCII^−^ for equine and CD90^+^, CD44^+^, CD45^−^ for canine AdMSCs. Only cells clearly characterized as AdMSC according to ISCT standards [26] were frozen and stored at the institute’s liquid nitrogen bank for further experiments. These characterizations for canine and equine AdMSC have already been published and will not be presented here [27, 28].

For the main experiments, passage 2 of canine and equine AdMSCs were rapidly thawed from the liquid nitrogen bank in a 37 °C water bath, centrifuged at 240×*g*, and placed in Dulbecco’s modified eagle’s medium, hereafter referred to as standard medium (DMEM, 31885023, Thermo Fisher Scientific, Germany) with low glucose supplemented with 10% fetal calf serum (FCS, lot. No. CP17-1688, Capricorn, Germany) and 1% penicillin/streptomycin (P/S, 15140122, Thermo Fisher Scientific, Germany) in an incubator at 37 °C and humid atmosphere. Prior to EVs generation and isolation, cells were passaged to obtain passage 3 cells and seeded in two 75 cm^2^ cell culture flasks (EasYFlasks™, Nunclon™Δ, 734-2066, VWR, Germany) per donor at a density of 10–15 k cells/cm^2^ containing 12 ml of standard culture medium.

### Conditioning of AdMSCs and downstream supernatant preparation

Once the cells were 80% confluent, they were first washed to ensure that nanoparticles in FCS did not interfere with the nanoparticles to be gained. For this, the medium in the cell culture flasks was replaced with 20 ml DMEM without FCS and the flasks were gently shaken on a shaker for 5 min. This washing procedure was repeated three times. Subsequently, 12 ml DMEM supplemented with 1% P/S and 1% insulin-transferrin-selenite solution (ITS-H, Capricorn, Germany) to compensate the absence of FCS was added to each cell culture flask. Three days later, the cell culture supernatant containing nanoparticles was harvested and centrifuged at 2700×*g* for 10 min at room temperature to remove cell debris. The supernatant was then filtered through a 0.2 µm syringe filter (83.1826.001, Sarstedt, Germany). The usual subsequent concentration process, e.g. ultracentrifugation, ultrafiltration or precipitation, was not performed in order to focus on the nanoparticles in the supernatant and the cryoprotectants without additional post-processing. Further purification and identification of nanoparticles containing equine small EVs, for example, has already been performed [29].

Since special attention was paid to short-term storage until additional concentration and/or experimental or even clinical application, no further processing of the nanoparticles obtained was carried out.

### Comparison of EDTA and TRE as freezing additives

Adipose tissue derived mesenchymal stem cells from 4 horses (mixed breeds and sexes, aged 15 ± 4.64 years) and 3 dogs (mixed breeds and sexes, aged 4.81 ± 3.51 years) were cultured to obtain nanoparticles as previously described. After obtaining 24 ml per donor of cell culture supernatant containing EVs, as described above, we divided it into 5 ml reaction tubes (Sarstedt, Germany), so that a total of 6 tubes with a volume of 4 ml from each donor were available for exposure to different storage conditions. These tubes were processed as follows: EDTA (Carl Roth, Germany) was added to obtain a final concentration of 5 mM (tube 1), TRE (Carl Roth, Germany) was added to three reaction tubes to obtain a final concentration of 25 µM (tube 2), 50 µM (tube 3) or 250 µM (tube 4), one tube as control of fresh (tube 5) and one tube as frozen samples (tube 6) without addition of TRE or EDTA. The size and concentration of EVs in the fresh reaction tubes (tube 5) were measured immediately after EVs purification by nanoparticle tracking analysis (NTA), whereas all other preparations were frozen at − 20 °C for 8 days (tubes 1–4, 6). After the freezing period, the reaction tubes were thawed in a 37 °C water bath and subsequently measured by NTA.

### Nanoparticle tracking analysis (NTA)

The nanoparticle tracking analysis can be used to track and characterize nanoparticles in a liquid with a size between 0.01 and 1 µm. For this purpose, the samples were diluted 1:1 with PBS (14190094, Thermo Fisher Scientific, Germany) and injected into the measurement chamber (NanoSight LM10, laser wavelength λ = 532 nm, Malvern Instruments Ltd., UK). Each sample was measured at three different liquid positions (three technical replicates) for 30 s at a temperature of 22 °C with a frame rate of 30 fps, a slider shutter of 1500 and a slider gain of 680. The captured videos were analyzed using the instrument manufacturers NTA analysis software (version 3.3, Malvern Instruments Ltd., UK) with a detection threshold of 5 and a maximal jump distance at an automatic setting with 9.5—9.9 pixel.

### Analysis of nanoparticles quality until 3 and 7 days of storage

Cell culture supernatants containing EVs were prepared as previously described from three horses (mixed breeds and sexes, aged 14 ± 4.97 years) and three dogs (mixed breeds and sexes, aged 4.81 ± 3.51 years). They were used for storage experiments for up to 7 days. 5 ml reaction tubes were filled with 2 ml supernatant and stored at 4 °C, 20 °C and 37 °C for 3 and 7 days with and without 5 mM EDTA. The concentration and size of EVs were then measured by NTA after 3 and 7 days of storage.

### Image generation

Images were generated using GraphPad Prism 7.05 (Figs. 1, 3, 5, 6 and 11) or were generated using the NTA analysis software version 3.3, Malvern Instruments Ltd., UK, merged with Adobe Photoshop CC (Figs. 2, 4, 7, 8, 9 and 10).

### Statistical analysis

To determine the effect of freezing agents including EDTA and TRE on the size and concentration of nanoparticles, an one way analysis of variance followed by the Holm–Sidak's multiple comparisons test was performed. To determine the influence of EDTA at different storage times (3 and 7 days) on the size and concentration of nanoparticles, a two way analysis of variance followed by Dunnett's multiple comparison test was applied. All values are presented as mean ± standard deviation. The statistical analysis and the figures were made using GraphPad Prism 7.05. The significance level was set at alpha ≤ 0.05.

## Results

### Comparison of EDTA and TRE as freezing agents on the EVs quality

#### Size of the EVs particles (nm)

**Fig. 1:**
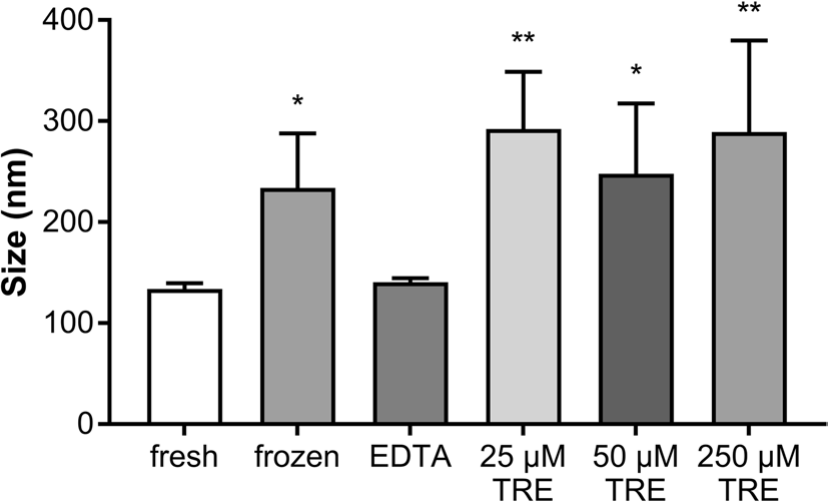
Size of EVs secreted by equine mesenchymal stem cells after freezing with and without addition of additives (n = 4, * P ≤ 0.05, ** for P ≤ 0.01)

After isolation and before freezing, the size of equine EVs could be determined as 134.37 ± 6.65 nm. Fresh addition of EDTA and TRE at the different concentrations mentioned above resulted in no detectable difference from the native sample (not shown further). Overall, freezing of EVs leads to an increase in particle size. Particle size within the frozen suspension without additives was increased after thawing to 233.93 ± 53.83 nm with a 1.7% increase in size. The addition of TRE in different concentrations (25, 50, 250 µM) after thawing even increased the particle size up to 292.25 ± 56.42 nm, 247.70 ± 69.86 nm and 289.1 ± 90.83 nm, respectively, with an average increase of 2%. In contrast, and as expected, the particle size of EVs was not altered by the addition of EDTA. Under these conditions, the size increased only slightly to 140.38 ± 4.13 nm. The differences were statistically compared with the particle size of freshly isolated EVs (control). No statistical difference could be calculated between fresh and EDTA-treated samples. The size of frozen particles was increased compared to fresh preparations (P ≤ 0.05). However, there was a significant change in particle size for samples treated with 25 µM TRE (P ≤ 0.01), 50 µM TRE (P ≤ 0.05) and 250 µM TRE (P ≤ 0.01) compared to fresh samples (Fig. 1).

**Fig. 2:**
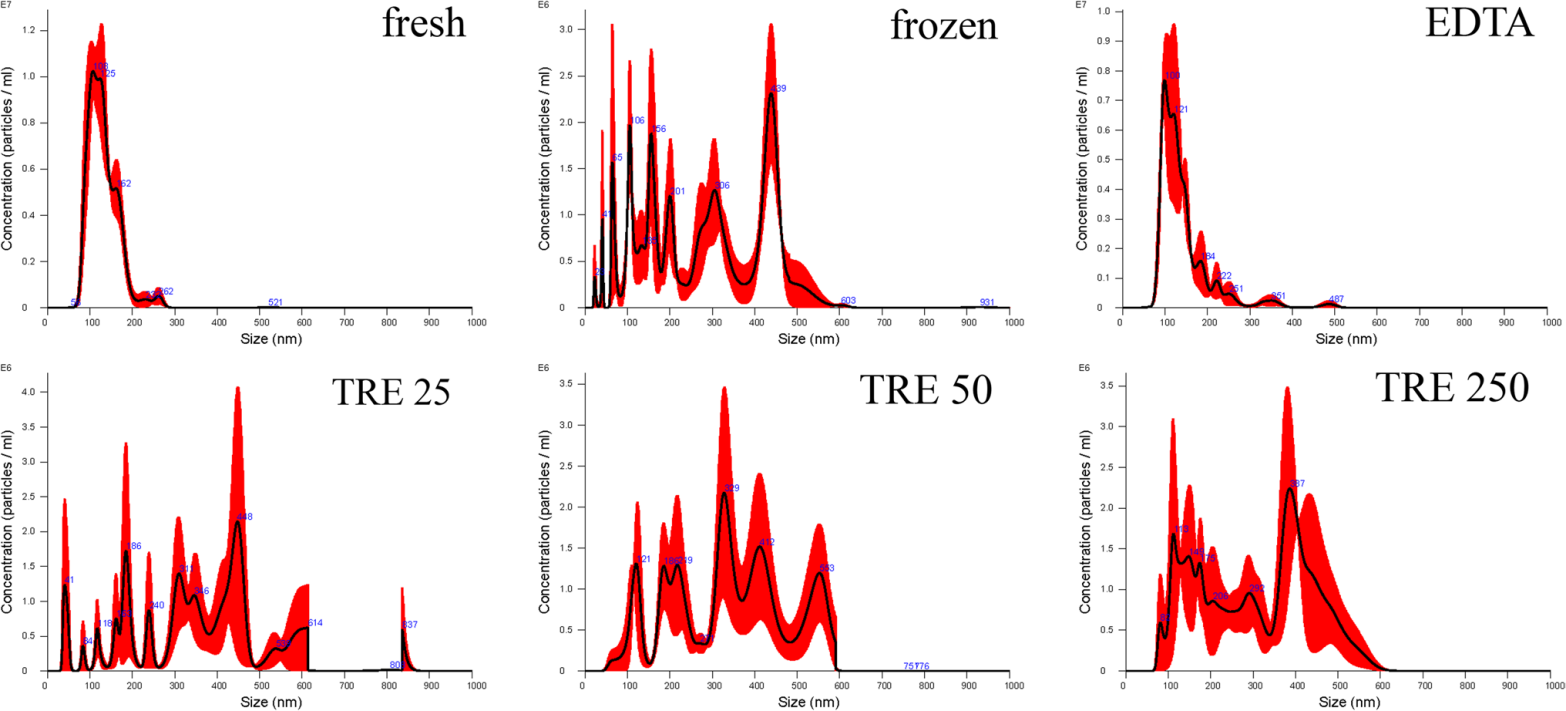
Size distribution of EVs secreted by equine mesenchymal stem cells from fresh (fresh), frozen without additives (frozen), frozen with addition of EDTA (EDTA), and frozen with addition of TRE at concentrations of 25 µM (TRE 25), 50 µM (TRE 50), and 250 µM (TRE 250)

The particle size distribution is shown in Fig. 2. It is clear to see how the particles aggregate once the EVs are frozen without the addition of additives or after the addition of TRE.

**Fig. 3:**
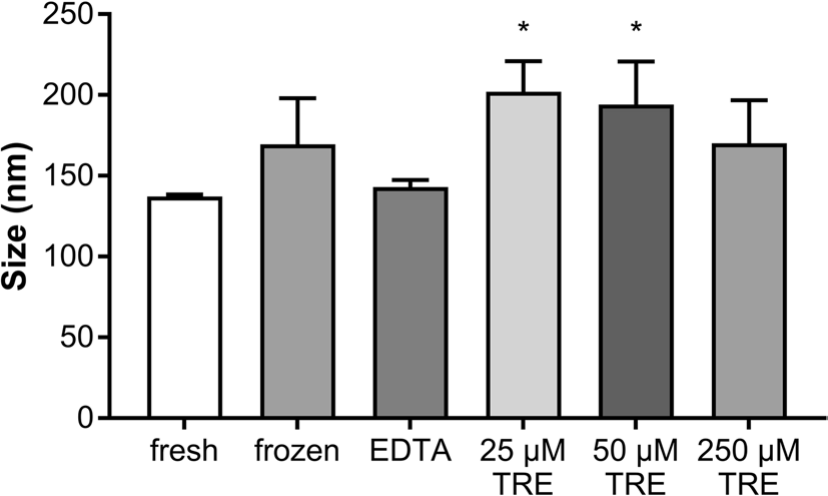
Size of EVs secreted by canine mesenchymal stem cells after freezing with and without addition of additives (n = 3, * indicates P ≤ 0.05)

The average size of fresh canine EVs analyzed directly after isolation was 137.13 ± 1.34 nm. After freezing, the size increased to 169.57 ± 28.54 nm. The addition of EDTA resulted in a comparable size of EVs to the fresh sample at 142.93 ± 4.52 nm. In contrast, the size of EVs supplemented with TRE was increased to 201.97 ± 19.02 nm, 194.13 ± 26.62 nm, and 170 ± 26.67 nm for the three TRE concentrations of 25 µM, 50 µM, and 250 µM, with an average increase in particle size of 1.4%. Moreover, there was an identical tendency as previously shown with equine derived EVs, only significant changes in particle size were detected with the addition of 25 µM TRE and 50 µM TRE concentrations (both P ≤ 0.05) compared to the fresh samples. Although the differences in EV size between frozen, EDTA-supplemented EVs and 250 µM TRE concentrations were not significant, a clear trend can be inferred (Fig. 3).

**Fig. 4:**
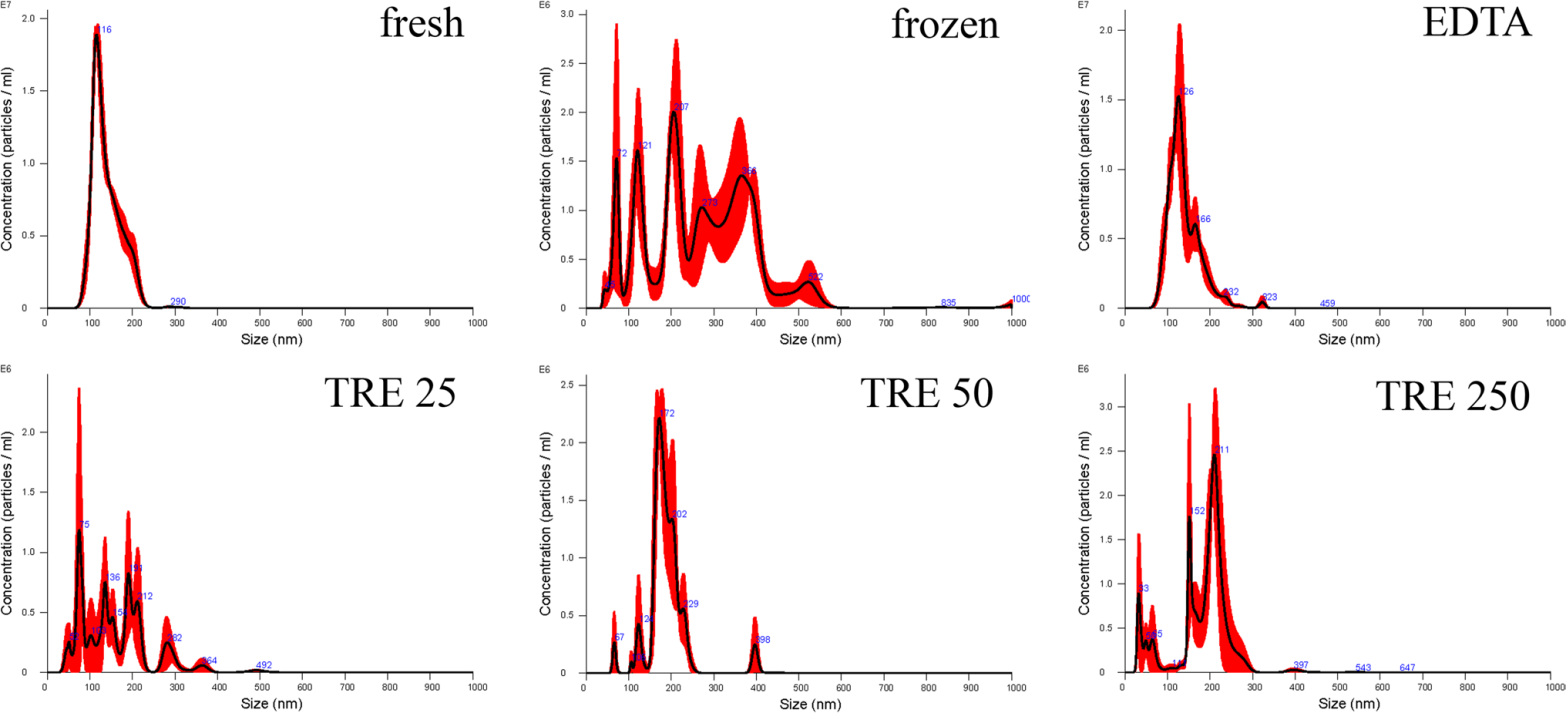
Size distribution of EVs secreted by canine mesenchymal stem cells from fresh (fresh), frozen without additives (frozen), frozen with addition of EDTA (EDTA), and frozen with addition of TRE at concentrations of 25 µM (TRE 25), 50 µM (TRE 50), and 250 µM (TRE 250)

As shown with equine EVs, canine EVs also tended to aggregate either in the absence of additives or when EVs were frozen with the addition of TRE (Fig. 4).

#### Number of EVs particles

**Fig. 5:**
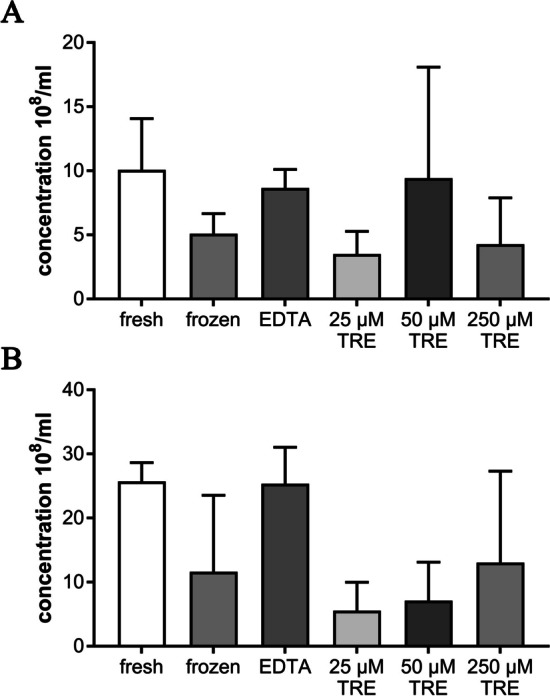
**A** Concentration of EVs secreted by equine mesenchymal stem cells after freezing with and without addition of additives (n = 4). **B** Concentration of EVs secreted by canine mesenchymal stem cells after freezing with and without addition of additives (n = 3)

With regard to the number of EVs determined under different storage conditions, there were no significant differences in the presence or absence of additives, only slight differences could be detected, resulting in a clear trend. Fresh supplementation of EDTA and TRE at the different concentrations resulted in no detectable difference from the native sample (not shown further). The highest amount of EVs could be detected immediately after their isolation (fresh) with numbers of 10.07 × 10^8^/ml and 25.73 × 10^8^/ml for equine and canine EVs, respectively. After treatment with 50 µM TRE for equine preparations, the concentration of EVs particles was 9.43 × 10^8^/ml (± 8.67). On the other hand, after treatment with EDTA for canine preparations, a concentration of 25.33 × 10^8^/ml (± 2.91) EVs could be detected. Other counts of equine EVs are 8.66 × 10^8^/ml (± 1.45) with EDTA, 5.10 × 10^8^/ml (± 1.56) for frozen without additives, 4.28 × 10^8^/ml (± 3.62) for 250 µM TRE and 3.51 × 10^8^/ml (± 1.77) for 25 µM TRE (Fig. 5A). Further counts of canine EVs were 13.05 × 10^8^/ml (± 14.28) for 250 µM TRE, 11.61 × 10^8^/ml (± 11.95) for frozen without additives, 7.12 × 10^8^/ml (± 6.00) for 50 µM TRE and 5.55 × 10^8^/ml (± 4.44) for 25 µM TRE (Fig. 5B). It was clearly observed that the concentration of EVs particles was reduced when treated with 25 µM and 250 µM TRE as shown in the equine isolated preparation. The same effect was more evident in canine isolated EVs after treatment with 25 µM, 25 µM and 250 µM TRE.

### Monitoring quality of EVs over a 7-day storage period

#### Size of the EVs particles (nm)

**Fig. 6:**
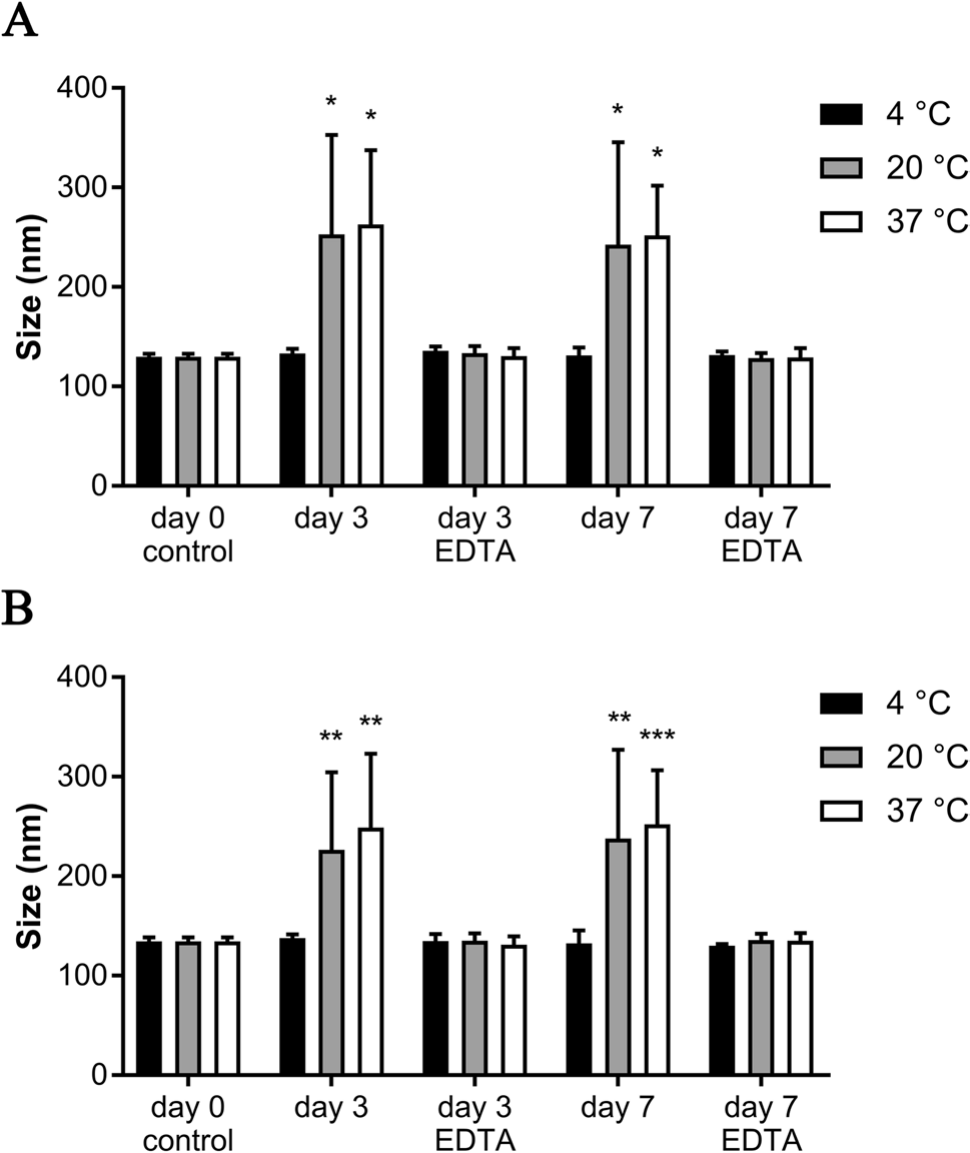
**A** Size of EVs secreted by equine mesenchymal stem cells after 0, 3- and 7-days of storage under different storage conditions (n = 3, * for P ≤ 0.05). **B** Size of canine mesenchymal stem cell secreted EVs (nm) after 0, 3-, and 7-days storage conditions (n = 3, ** for P ≤ 0.01, *** for P ≤ 0.001)

The quality (size and concentration) was measured over a period of 7 days on days 0, 3 and 7. Here we could clearly show that the use of EDTA prevented the EVs from clumping and therefore maintained their natural distribution when not refrigerated at 4 °C. Similar to the addition of EDTA, chilling effectively prevented aggregation of EVs. Specifically, fresh equine EVs had a size of 129.67 ± 3.17 nm, whereas canine EVs had a size of 134.37 ± 4.19 nm before storage (Fig. 6A, B).

In addition, a significant increase in particle size was observed for equine EVs after storage at room temperature for 3 days (252.67 ± 100.26 nm, P = 0.02) and 7 days (242.57 ± 102.88 nm, P = 0.03). The same results were observed after storage at 37 °C, with a significant increase in particle size at days 3 and 7 (P = 0.01 and P = 0.02) compared to fresh preparations. Analysis of canine EVs showed a similar effect in terms of increased particle size after 3 (P = 0.009) and 7 days (P ≤ 0.01) of storage at room temperature. Storage at 37 °C showed an increase in particle size after 3 (P = 0.001) and 7 days (P = 0.003) compared to fresh isolation (control). All results, including the remaining insignificant results (P > 0.05), are shown in Tables 1 and 2.

**Table 1:** Sizes (nm) of equine EVs after storage under various condition (${\overline{\text{x}}}$ ± SD)

Storage conditions	Day 0	Day 3			Day 7		
		4 °C	20 °C	37 °C	4 °C	20 °C	37 °C
W/o EDTA	129.67 ± 3.17	133.17 ± 3.17	**252.60** ± 100.26	**262.77** ± 74.70	131.10 ± 7.97	**242.57** ± 102.88	**251.97** ± 49.80
With EDTA	n/a	135.70 ± 4.57	133.37 ± 7.10	130.40 ± 7.95	131.40 ± 3.67	128.37 ± 5.10	129.17 ± 9.45

Mean values in bold indicate a significant change

**Table 2:** Sizes (nm) of canine EVs after storage under various condition (${\overline{\text{x}}}$ ± SD)

Storage conditions	Day 0	Day 3			Day 7		
		4 °C	20 °C	37 °C	4 °C	20 °C	37 °C
W/o EDTA	134.37 ± 4.19	137.70 ± 3.66	**226.60** ± 77.87	**248.93** ± 74.26	132.63 ± 12.89	**237.87** ± 89.39	**252.03** ± 54.40
With EDTA	n/a	134.70 ± 7.05	135.27 ± 7.20	131.20 ± 8.32	130.10 ± 1.61	135.97 ± 6.15	135.13 ± 7.85

Mean values in bold indicate a significant change

**Fig. 7:**
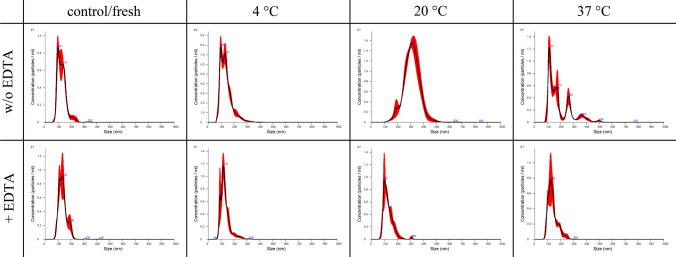
Comparison of the size distribution of EVs secreted by equine mesenchymal stem cells with (+EDTA) and without (w/o EDTA) addition of EDTA under different storage conditions (refrigerated at 4 °C, room temperature at 20 °C, incubator at 37 °C) after 3 days of storage

**Fig. 8:**
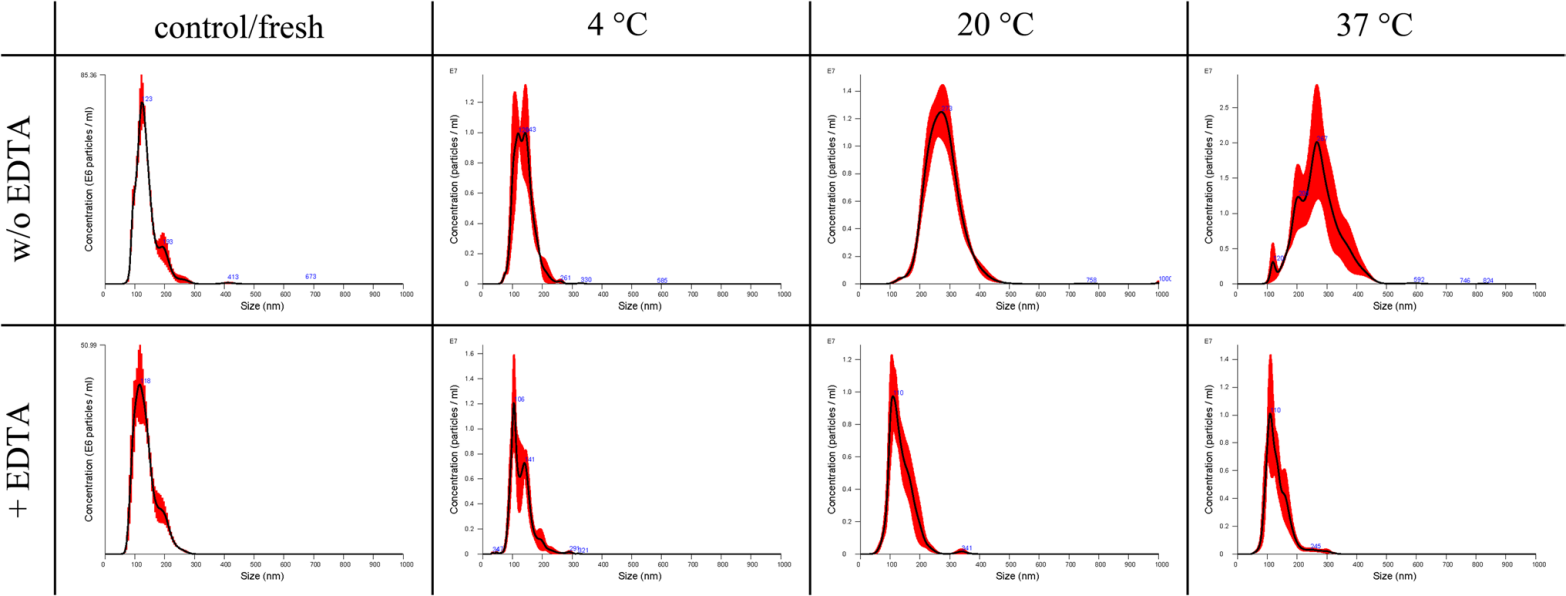
Comparison of the size distribution of EVs secreted by canine mesenchymal stem cells with (+EDTA) and without (w/o EDTA) addition of EDTA among different storage conditions (refrigerated at 4 °C, room temperature at 20 °C, incubator at 37 °C) after 3 days of storage

**Fig. 9:**
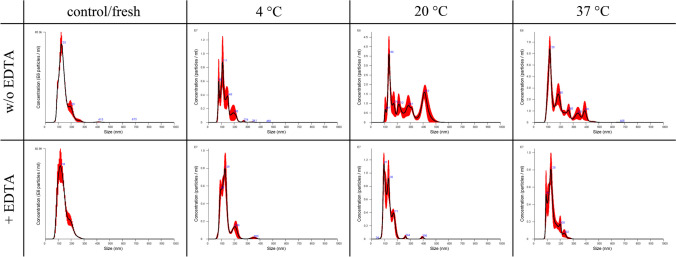
Comparison of the size distribution of EVs secreted by equine mesenchymal stem cells with (+EDTA) and without (w/o EDTA) addition of EDTA among different storage conditions (refrigerated at 4 °C, room temperature at 20 °C, incubator at 37 °C) after 1 week of storage

**Fig. 10:**
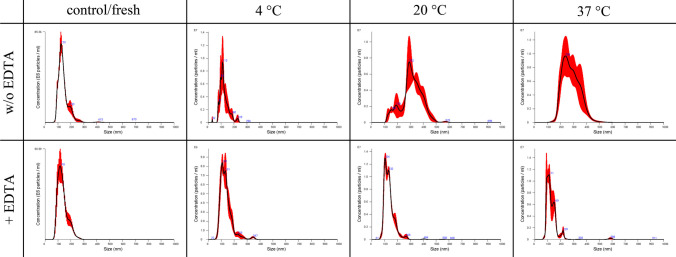
Comparison of the size distribution of EVs secreted by canine mesenchymal stem cells with (+EDTA) and without (w/o EDTA) addition of EDTA under different storage conditions (refrigerated at 4 °C, room temperature at 20 °C, incubator at 37 °C) after one week of storage

The size distribution during different storage conditions after 3 (Figs. 7 and 8) and 7 days (Figs. 9 and 10) for equine and canine secreted EVs visually underlines the cryoprotective properties of EDTA.

#### Number of EVs

**Fig. 11:**
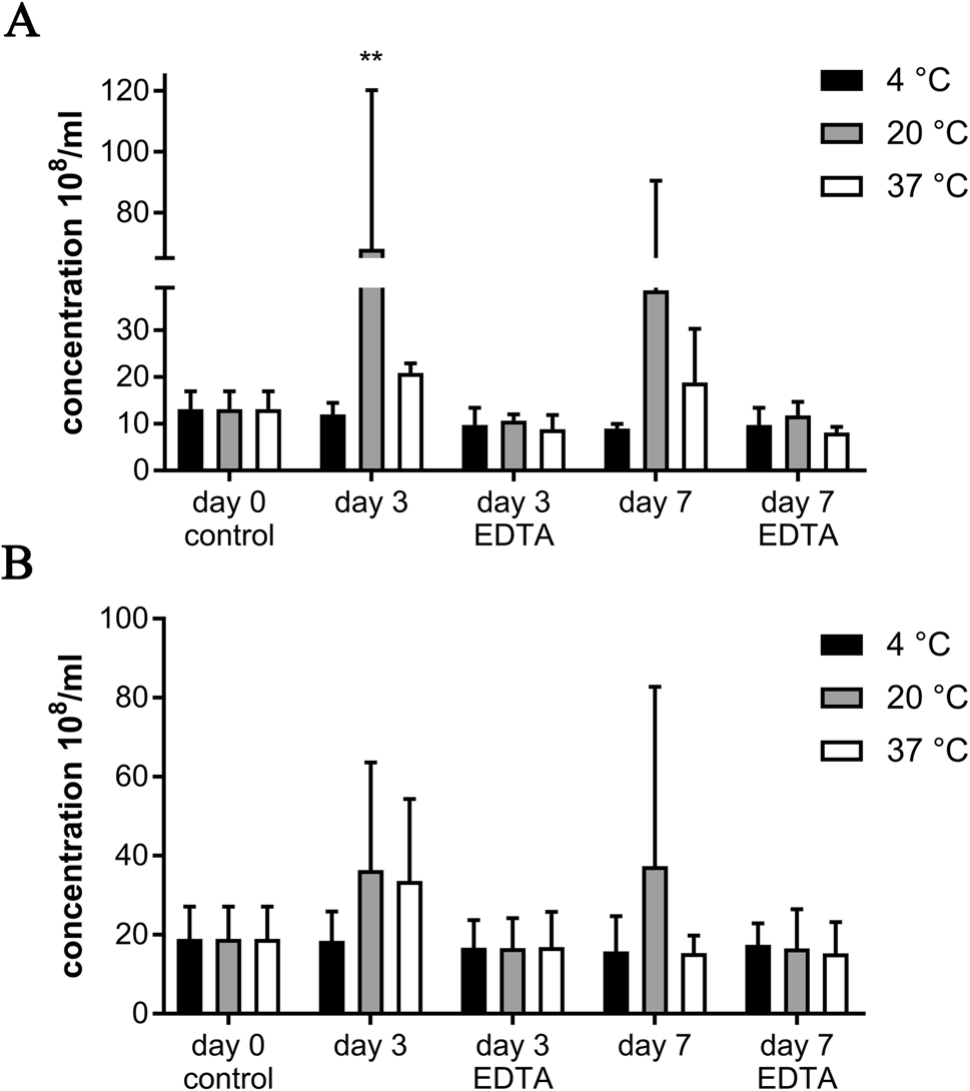
**A** Concentration of EVs secreted by equine mesenchymal stem cells after 0, 3- and 7-days storage under different storage conditions (n = 3). **B** Concentration of EVs secreted by canine mesenchymal stem cells after 0, 3-, and 7-days storage after different storage conditions (n = 3)

At day 0, the concentration of EVs was 13.11 × 10^8^/ml ± 3.76 and 18.93 × 10^8^/ml ± 8.17 for equine and canine, respectively (Fig. 11A, B).

While the concentration of equine EVs was slightly increased after 3 days of storage without addition of EDTA at 20 °C (P = 0.009) and 37 °C (P > 0.05), canine EVs did not show such performance even after 7 days of incubation at 37 °C. However, we could see a slight increase after 7 days at 20 °C (P = 0.45). After 3 days of storage the concentration of canine EVs at 20 °C and 37 °C increased slightly without statistically significant changes (P = 0.55 and P = 0.73). All results, including the remaining insignificant results (P > 0.05), are shown in Tables 3 and 4.

**Table 3:** Concentration (10^8^/ml) of equine EVs after storage under various conditions (${\overline{\text{x}}}$ ± SD)

Storage conditions	Day 0	Day 3			Day 7		
		4 °C	20 °C	37 °C	4 °C	20 °C	37 °C
W/o EDTA	13.11 ± 3.76	11.97 ± 2.47	**68.11** ± 52.04	20.81 ± 2.07	8.93 ± 1.04	38.51 ± 51.92	18.83 ± 11.44
With EDTA	n/a	9.73 ± 3.65	10.65 ± 1.34	8.83 ± 2.99	9.70 ± 3.70	11.75 ± 2.87	8.14 ± 1.17

Mean value in bold indicate a significant change

**Table 4:** Concentration (10^8^/ml) of canine EVs after storage under various conditions (${\overline{\text{x}}}$ ± SD)

Storage conditions	Day 0	Day 3			Day 7		
		4 °C	20 °C	37 °C	4 °C	20 °C	37 °C
W/o EDTA	18.93 ± 8.17	18.42 ± 7.44	36.35 ± 27.24	33.61 ± 20.80	15.77 ± 8.93	37.40 ± 45.40	15.35 ± 4.41
With EDTA	n/a	16.67 ± 7.06	16.66 ± 7.57	16.85 ± 8.92	17.49 ± 5.38	16.51 ± 9.98	15.25 ± 7.93

## Discussion

Extracellular vesicles provide a valuable tool to study intercellular communication and disease progression, such as cancer metastasis [30]. Isolation of EVs for long-term storage is an important consideration, especially when capacities are limited to obtain sufficient quantities for either therapeutic applications or basic research. Typically, various substances are stored by refrigeration at 4 °C and freezing at − 20 °C for limited use or even lower temperatures at − 196 °C for long-term storage. Freezing and cooling are often aided by the addition of cryoprotectants including dimethyl sulfoxide (DMSO) [22, 31], EDTA [32] (anticoagulant) and TRE for EVs stabilization [19], which support and stabilize the frozen/cooled substances by various mechanisms.

In the present study, we evaluated the influence of EDTA and TRE on the properties of EVs after storage at room temperature (20 °C), refrigerated (4 °C), in an incubator (37 °C), and frozen at − 20 °C. These temperatures can be maintained in many laboratories or clinics under standard conditions and are an important assessment to understand whether long-term storage of EVs is possible in principle without loss of viability and functional performance. Previous reports have concluded that the homogeneous distribution of EVs promotes their stability during storage and preserves their function [33]. Our data showed that after freezing, the formation of EV aggregates could be detected, as shown by an increased size distribution of the particles. This clearly indicates that the recovered EVs had a tendency to aggregate with each other after freezing, probably due to changes in surface structures. Thus, our data suggest that freezing could modulate the surface charges and antigens of the EV membrane. This effect would alter the interaction of EVs and cause their aggregation. It has been reported that the surface charges of EVs are regulated by their zeta potential. Increased zeta potential creates electrostatic repulsion between individual EVs and reduces their aggregation [34]. In addition, and in the same line of reasoning, a study showed that aggregation of EVs occurs under several conditions, including the extraction process, freezing and thawing, and storage [35]. Furthermore, either freezing or lyophilization could also reduce the bioactivity of extracellular vesicles after hypoxia-induced injury to muscle cells [36].

The mechanism of action of TRE, a commonly used cryoprotectant, is to minimize intercellular ice crystal formation during freezing to reduce protein aggregation [37]. In addition, it was also shown that the buffer used is of greater importance. In particular, Goerges et al. [21] investigated that the sole use of PBS is not suitable for storing EVs over a longer period of time. Instead, they were able to show that the addition of HEPES, albumin and trehalose is best suited for this purpose. Especially when several freezing and thawing cycles are carried out. Our data showed that the addition of TRE did not prevent the expected aggregation of EVs. In fact, a study by Budgude et al. [38] also showed that the size of the EVs increases when stored for over a month with the addition of TRE, even significantly at a temperature of 4 °C. Only when stored in liquid nitrogen (− 196 °C) did the size decrease. This effect may be dependent on the time frame between sample isolation and processing prior to the addition of TRE. In addition, the concentration used may be higher than that required to prevent aggregation. In contrast to our data, a concentration of 25 mM TRE was found to be sufficient not only to protect the morphology, RNA and protein content of EVs after freezing and thawing, but also to maintain the number of particles [19]. A similar study examined the size of EVs isolated from melanoma cells after lyophilization at room temperature compared to preservation at − 80 °C. The authors found that treatment with trehalose prevented EV aggregation compared to lyophilization without the addition of a cryoprotectant [20]. Moreover, the addition of trehalose as a cryoprotectant to the frozen dried extracellular vesicles was able to increase the viability of muscle cells after hypoxia [36]. However, in the present study, the EVs-containing supernatant of AdMSCs was combined with different concentrations of trehalose and without lyophilization.

The results showed that the addition of 5 mM EDTA resulted in an almost identical size distribution after thawing compared to the fresh preparation. These observations were expected because EDTA is widely used as an anticoagulant and may play a role in reducing EV aggregation and maintaining homogeneous dispersion of EV particles in the buffer. It has been reported that anticoagulants such as EDTA and citrate reduce or even eliminate microvesicles from plasma by increasing their attachment to platelets. However, the addition of heparin was able to maintain the number of microvesicles [39].

Although previous studies have shown that long-term storage under either lyophilization or freezing reduces the integrity of EVs and even their bioactivity [40]. In fact, our data showed that refrigeration of EVs at 4 °C for 7 days did not cause any change in the size and concentration of EVs particles, even without any cryoprotective agent. Short storage without additives at 4 °C had also been shown by Cheng et al. in HEK 293 T cells [41]. Moreover, the data suggest that low refrigeration for up to 7 days without any precipitating agent could maintain EVs dispersion and is therefore a possible storage target for short-term application. On the other hand, the present study showed an increased size of EVs particles at day 3 and 7 after incubation at 20 °C and 37 °C for both equine and canine preparations. The possible explanation could be that EVs tend to aggregate at higher temperatures during the storage period, which increased the size of the measured particles as shown by NTA. In the same line, it has been reported that the proteins associated with the extracellular vesicles are sensitive not only to the storage temperature but also to the time span before the preparation is processed. [42]. Typically, freezing at − 80 °C has been the most widely used method for long-term storage. In this regard, a previous study has documented that freezing at − 80 °C for semen-derived extracellular vesicles maintains their morphology, size, and concentration for up to 30 years [43]. Moreover, and in agreement with our data, a patent examined EVs from cardiosphere-derived cells and concluded that extracellular vesicles are stable in size more than 7 days of storage at 4 °C, − 20 °C and − 80 °C as shown by NTA. Interestingly, however, bioactivity, as indicated by miRNA content, was reduced by up to 50% after 30 days of storage at only 4 °C and − 20 °C [44]. However, the addition of DMSO as a classic cryoprotectant—essentially for living cells—has also been shown to cause morphological changes after storage at − 80 °C, as well as changes in the RNA it contains. This suggests that EVs mixed with DMSO no longer have the same qualitative properties as freshly obtained EVs [22].

The data showed a reduction in the number of EVs when treated with 25 µM and 250 µM TRE in equine preparations and a much greater reduction in all TRE concentrations in dogs compared to fresh samples. These data are in parallel with the increased size of the EVs particles as mentioned previously and suggest that the aggregation of EVs when TRE was used resulted in a reduction in particle count. This also indicates that no protein degradation occurred for the isolated EVs. In contrast, analysis showed that the number of EVs remained stable with 5 mM EDTA treatment, similar to fresh preparations for both equine and canine EVs. These data are consistent with previous results showing no changes in particle size, as no aggregation could be detected, in contrast to the observation with supplemented TRE. In addition, the data suggest that a concentration of 5 mM EDTA is suitable for storage of EVs at 4 °C, 20 °C and 37 °C for up to one week. It is well known that EDTA acts as a chelator, which may interact with the surface proteins or even ions in the plasma membrane of EVs, resulting in inhibited aggregation. The most convincing explanation is that Ca^2+^ ions, chelated by EDTA and thus inaccessible, prevent EVs adhesion proteins from interacting with other EVs and thus inhibit aggregation in the liquid buffer, thus maintaining their dispersibility. In addition, it has been reported that N-cadherin is dependent on Ca^2+^ to establish stable adhesion to the extracellular matrix [45, 46], and Ca^2+^ depletion causes a sudden loss of cell adhesion and intercellular junction [47]. In addition, N-cadherin was found to play an important role in cell aggregation in suspensions, and downregulation of N-cadherin using EDTA-trypsin delayed cell aggregation [48]. This could be a part of the reason why the size of the EVs remains stable after storage with EDTA.

## Conclusion

Canine and equine mesenchymal stem cell-derived EVs can be stored at 4 °C for 7 days without addition of cryoprotectants without changes in size and concentration. If EVs need to be frozen, the addition of EDTA is appropriate to prevent changes in size and therefore characteristics of EVs for at least one week under all storage conditions. In contrast, based on our data, trehalose is not suitable for maintaining the stability of EVs during storage of EVs enriched supernatant from conditioned AdMSCs.

## Data Availability

The datasets used and analysed during the current study are available from the corresponding author on reasonable request.

## Abbreviations

EVs: Extracellular vesicles
AdMSCs: Adipose tissue derived mesenchymal stem cells
DMSO: Dimethyl sulfoxide
MSCs: Mesenchymal stem cells
EDTA: Ethylenediaminetetraacetic acid
TRE: Trehalose
FCS: Fetal calf serum
P/S: Penicillin/streptomycin
NTA: Nano tracking analysis
